# Will the Meikirch Model, a New Framework for Health, Induce a Paradigm Shift in Healthcare?

**DOI:** 10.7759/cureus.1081

**Published:** 2017-03-06

**Authors:** Johannes Bircher, Eckhart G Hahn

**Affiliations:** 1 Hepatology, University of Berne, Switzerland; 2 Department of Medicine 1, University Hospital Erlangen

**Keywords:** definition of health, meikirch model, value-based health care, new health policies, potentials for health, health as a complex adaptive system, responsibility for health, social determinants of health, environmental determinants of health, life´s demands

## Abstract

Over the past decades, scientific medicine has realized tremendous advances. Yet, it is felt that the quality, costs, and equity of medicine and public health have not improved correspondingly and, both inside and outside the USA, may even have changed for the worse. An initiative for improving this situation is value-based healthcare, in which value is defined as health outcomes relative to the cost of achieving them. Value-based healthcare was advocated in order to stimulate competition among healthcare providers and thereby reduce costs. The approach may be well grounded economically, but in the care of patients, “value” has ethical and philosophical connotations. The restriction of value to an economic meaning ignores the importance of health and, thus, leads to misunderstandings.

We postulate that a new understanding of the nature of health is necessary. We present the Meikirch model, a conceptual framework for health and disease that views health as a complex adaptive system. We describe this model and analyze some important consequences of its application to healthcare.

The resources each person needs to meet the demands of life are both biological and personal, and both function together. While scientific advances in healthcare are hailed, these advances focus mainly on the biologically given potential (BGP) and tend to neglect the personally acquired potential (PAP) of an individual person. Personal growth to improve the PAP strongly contributes to meeting the demands of life. Therefore, in individual and public health care, personal growth deserves as much attention as the BGP. The conceptual framework of the Meikirch model supports a unified understanding of healthcare and serves to develop common goals, thereby rendering interprofessional and intersectoral cooperation more successful. The Meikirch model can be used as an effective tool to stimulate health literacy and improve health-supporting behavior. If individuals and groups of people involved in healthcare interact based on the model, mutual understanding of and adherence to treatments and preventive measures will improve. In healthcare, the Meikirch model also makes it plain that neither pay-for-performance nor value-based payment is an adequate response to improve person-centered healthcare.

The Meikirch model is not only a unifying theoretical framework for health and disease but also a scaffold for the practice of medicine and public health. It is fully in line with the theory and practice of evidence-based medicine, person-centered healthcare, and integrative medicine. The model offers opportunities to self-motivate people to improve their health-supporting behavior, thereby making preventive approaches and overall healthcare more effective.

We believe that the Meikirch model could induce a paradigm shift in healthcare. The healthcare community is hereby invited to acquaint themselves with this model and to consider its potential ramifications.

## Introduction and background

Although healthcare systems worldwide accomplish more than ever, they are neither as successful nor as sustainable as they could be [1]. Costs are increasing at a greater rate than the gross domestic products of nations, and patients, as well as many healthcare providers, are more and more discouraged with the burdens of practice; the trust of patients in their physicians seems to be decreasing [2]. Newer management approaches define value as “health outcomes relative to the cost of achieving them” [3]. As a result, economic considerations appear to have become as important as health itself as an outcome. A central reason for these conflicting trends may relate to how we think about health. In discussions about healthcare, the term “health” seems to be ignored (a “blind spot”). In our current conception, health is realized only when symptoms, signs, or functional limitations occur. This means that awareness of health occurs when it has already been compromised or lost. Therefore, in current thinking, health may be described in a negative connotation, and until recently, a satisfactory concept of health in its own right has been unavailable. In article 12 of the International Covenant on Economic, Social and Cultural Rights of the World Health Organization (WHO), health was described as a human right, and determinants of health were identified as “the highest attainable standard of physical and mental health” [4]. In this ground-breaking document, the definition of health contained in the preamble to the Constitution of WHO, which conceptualizes health as “a state of complete physical, mental and social well-being and not merely the absence of disease or infirmity,” was actually not adopted. Several concepts of health have subsequently been suggested [5], none of which has so far been accepted and successfully applied to ascertain the health of individuals and public health.

This deficit was recently addressed by the introduction of the Meikirch model [5], in which a definition of health describes health on a meta-level by its structure and functions. The model uses a framework of health consisting of five components that are related to each other by 10 complex interactions. This concept has received attention because it allows health and disease to be observed and analyzed in a new context and with new consequences [6]. The model has also been explored for its applicability in the practice of healthcare [7]. The purpose of the present paper is to review the Meikirch model and to investigate its possible role in a paradigm change in an era of value-based healthcare.

## Review

### The Meikirch model

Origin of the Name and Graphic Representation

**Figure 1:**
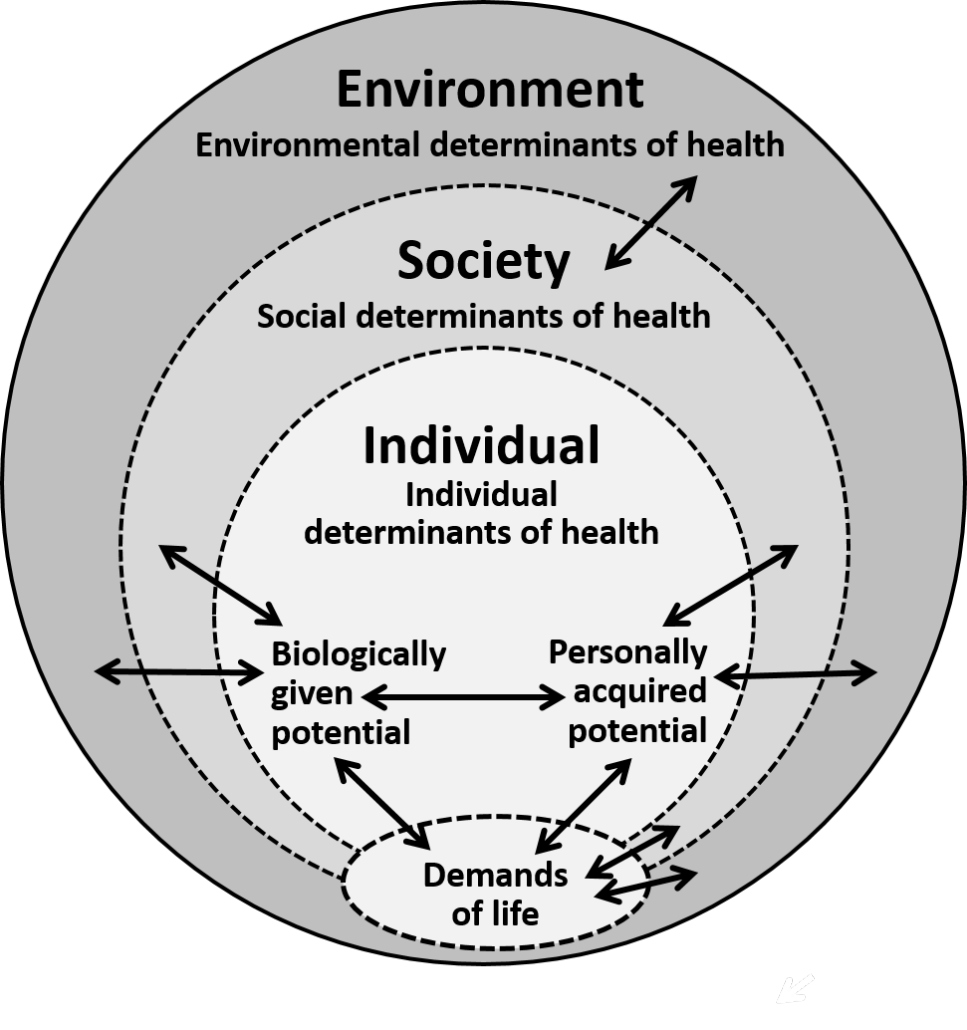
The Meikirch model The Meikirch model consisting of five components: The demands of life, an individual’s two potentials, and the social and environmental determinants of health. The double arrows express ten complex interactions between these components. (Figure originates from reference 6)

This new definition of health has been referred to as the Meikirch model. Meikirch is a village in Switzerland where the model was first conceived. A graphical representation of the model is shown in Figure 1.

Wording of the Model

“Health is a dynamic state of well-being emergent from conducive interactions between an individual’s potentials, life’s demands, and social and environmental determinants. Health results throughout the life course when an individual’s potentials – and social and environmental determinants – suffice to respond satisfactorily to the demands of life. Life’s demands can be physiological, psychosocial, or environmental and vary across individuals and contexts but, in every case, unsatisfactory responses lead to disease” [5-6].

Demands of Life

For survival, every living creature must satisfy its respective demands of life. This is a general biological postulate that varies from species to species and applies also to man. In humans, these demands may be categorized as physiological, psychosocial, and environmental.

Potentials

**Figure 2:**
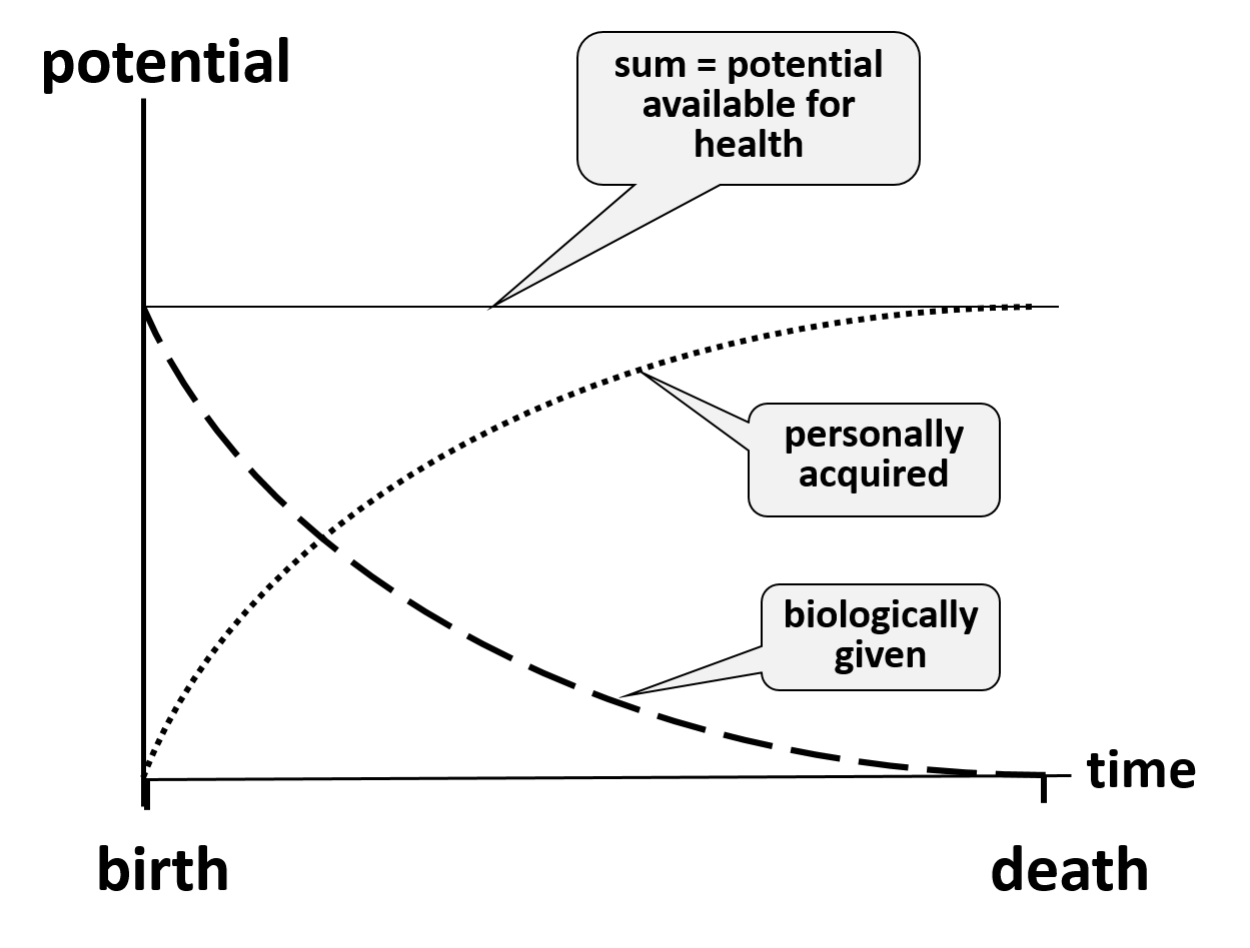
The biologically given potential and the personally acquired potential Graph showing an idealized time course of the two potentials. The biologically given potential has a finite value at the time of birth and thereafter decreases continuously throughout life and reaches zero when the person dies. The personally acquired potential is small at the time of birth, increases rapidly shortly thereafter, and later increases more slowly. It may grow throughout life provided that the individual assumes the responsibility of caring for it. During a crisis or because of alcohol or drug addiction, it may decrease transiently or permanently. When a person is challenged by the demands of life, a satisfactory response always involves both potentials. Throughout the life course, the contribution of each potential to the sum, i.e., to the health of an individual, varies continuously, and therefore, attention to personal growth is important. (Figure derived from reference 30)

**Figure 3:**
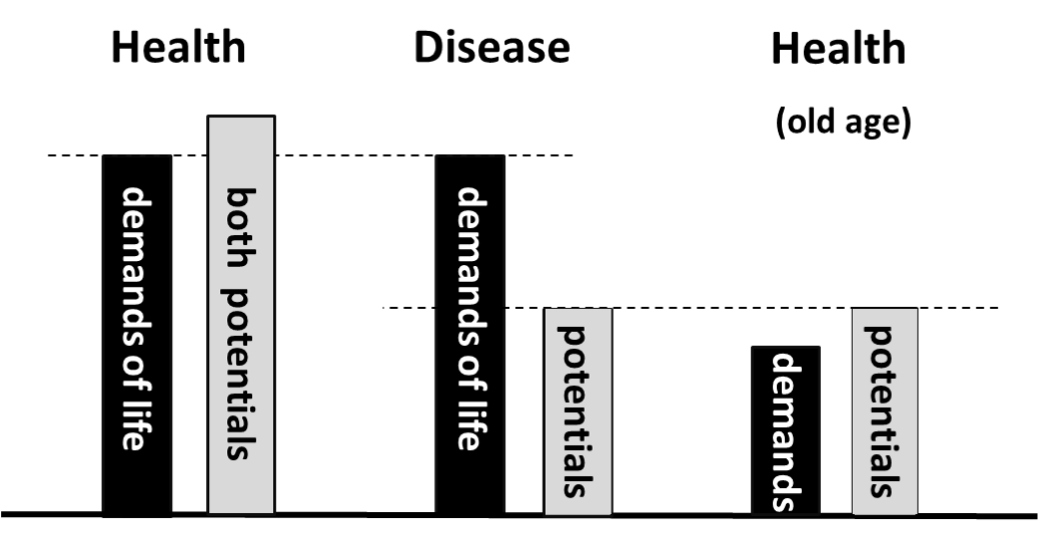
Distinction between health and disease Health results (left) whenever the two potentials together are larger than that needed to satisfy the demands of life. When they are smaller than the demands of life (middle), disease occurs. When the potentials are reduced, health may result again, provided the demands of life have decreased even more (right). In this case, the subjects usually say that they are healthy.

Health requires that individual resources match the demands of life. This means that every person must be able to respond satisfactorily to the demands of life and possible changes. Since these resources are needed not only at a specific time but also in the long-term future, they are called potentials. Each individual is equipped with two types of potentials, a biologically given potential (BGP) and a personally acquired potential (PAP). The BGP is the gift of nature everyone receives at the time of birth. It represents the biological basis of human existence. After birth, this potential decreases continuously and reaches zero at the time of death (Figure 2). Diseases and accidents may reduce it transiently or permanently (Figure 3). The term PAP is used to describe all the physical, mental, and spiritual abilities an individual can acquire during his or her lifetime. It is also the site of individual responsibility for health. After birth, the PAP increases rapidly. Thereafter, growth slows; it may continue, though, if a person invests in the development of abilities and inner growth. Efforts to develop the PAP are important investments in an individual´s future health. In a personal crisis, the PAP may decrease but recover, more or less, once the crisis is overcome. For example, alcohol or drug addiction will decrease the PAP, transiently or for life. In responding to the demands of life, the two potentials always act together. Notably, the PAP can compensate to some degree for deficiencies in the BGP. This is particularly needed as a person ages.

The two potentials continuously interact with each other. This interaction may be explained best with an analogy: If a rider wants her horse to serve her well, she must take good care of her horse in every respect. For a horse to be well, it needs fluids and food, cleanliness, physical activity, a safe place to rest, and sufficient personal attention by the rider. While giving attention to the horse, the rider must stay in control and must not allow the horse to take over.

Social Determinants of Health

As shown in Figure 1, the individual determinants of health are immersed in and surrounded by social determinants of health. Initially, it is the mother who takes care of the newborn. Thereafter, the whole family, the school, and professional education become important. Later, individuals interact in multiple ways with their social environment, which becomes the concern of public health. The social determinants interact also with the “demands of life” and thereby modify, for example, working conditions and the demands of life. All these interactions influence the potential to respond satisfactorily to the changing demands of life for each individual. Obviously, the contribution of social determinants must be conducive to the BGP, PAP, age, and specific social setting of an individual. This is a responsibility that society must fulfill.

Environmental Determinants of Health

In Figure 1, the outer ring corresponds to the environmental determinants of health. These vary with the geographical location of a person, the local situation, and the amount of pollution in the environment. For instance, in Switzerland (and elsewhere), there is insufficient iodine in the natural surroundings. Table salt is thus now iodinated, and goiters and cretinism have practically disappeared. The environmental determinants interact with the social determinants of health, the BGP, the PAP, and the demands of life.

Complex Adaptive System

The Meikirch model consists of five components and ten complex interactions (Figure 1). In systems theory, such an organization corresponds to a complex adaptive system (CAS), a term in science that comprises a number of interesting properties [8-10]. The overall performance of a CAS cannot be deduced from its parts because it exhibits qualities, referred to as emergence, that are different from and much more than the sum of its parts. For example, human thinking, consciousness, and creativity are qualities that cannot be predicted from an analysis of organs or cells. What this means for traditional scientific medicine and person-centered care is detailed in Table 1.

**Table 1:** Comparison of Thinking in Traditional Scientific Medicine and in Person-centered Care *Value is defined as the health outcomes achieved for patients relative to the costs of achieving them.

Thinking	Traditional medicine	Person-centered care
Methods	Newtonian science	Complexity science
Meikirch model	Biologically given potential	Personally acquired potential
Health	Proper function of organs and lack of symptoms	Well-being, life satisfaction
General results of interventions	Predictable cause-effect relationships	Unpredictable, combined with strong resistance toward manipulation
Medicine	Imaging, clinical chemistry, microbiology, drug treatment, surgery	Family medicine, psychotherapy, rehabilitation, complementary medicine
Outcome	Predictable, measurable	Unpredictable
Public health	Protection at the workplace, water hygiene, safety of nutrients, vaccinations, etc.	Support for families and schools, care for personal growth, healthy work-life balance, healthy aging, palliative care, etc.
Payment system	Spectrum from fee-for-service to value-based payment*	Methods that support devotion to patient care irrespective of the desired outcome

The identification of health as a CAS leads to a new understanding of properties that are most pertinent to healthcare. All of them must be considered when trying to understand health and disease. A CAS always functions as a whole and adapts autonomously to changes in its surroundings. The success of such an adaptation varies from time to time, from situation to situation, and from CAS to CAS. Manipulation of the system by medical doctors may be successful for a BGP, which is part of the CAS, as occurs with joint replacement, coronary artery stenting, or pharmacotherapy. Yet, a CAS intensely resists external guidance or manipulation. Top-down management of a CAS does not work. However, autonomous gradual evolution is one of its intrinsic properties that results from its existence close to the frontier between chaos and order. A chaotic state, by nature, remains chaotic and, as such, does not evolve. On the other hand, a fully ordered state corresponds to a machine that does not evolve either. Only when a system can fluctuate close to the interface between order and chaos can it gradually adjust on its own to changing circumstances.

Chaos may lead to random changes that may be useful or detrimental. In the former case, the changes are explorative, and in the latter, they may be considered to be mistakes. Order can integrate both types of changes into the system, thereby rendering it more or less adapted to its surroundings. With such an arrangement, a CAS may respond successfully to new challenges and evolve toward new properties. This represents personal development or growth. Sometimes, it is surprising to observe how much an individual can adjust to difficult life situations. At other times, rigidity may be an obstacle to progress. In man, loving relationships, creativity, positive feelings, recognition of a purpose in life, and psychotherapy are factors that tend to support favorable evolutions [11]. This was well expressed by Antonovsky as a sense of coherence [12]. He proposed that for health an individual must fully understand the conditions of his or her life, be able to handle problems well, and feel that activities for his or her health are purposeful. For example, a patient with Type 1 diabetes mellitus must understand the glucose and insulin physiology, be able to measure blood glucose levels and inject insulin, and feel that careful treatment of his or her condition makes sense. To achieve this, contributions from both the PAP and the society are needed.

### Application of the Meikirch model in healthcare and public health

Biology-centered and Person-centered Healthcare are Closely Interrelated

**Figure 4:**
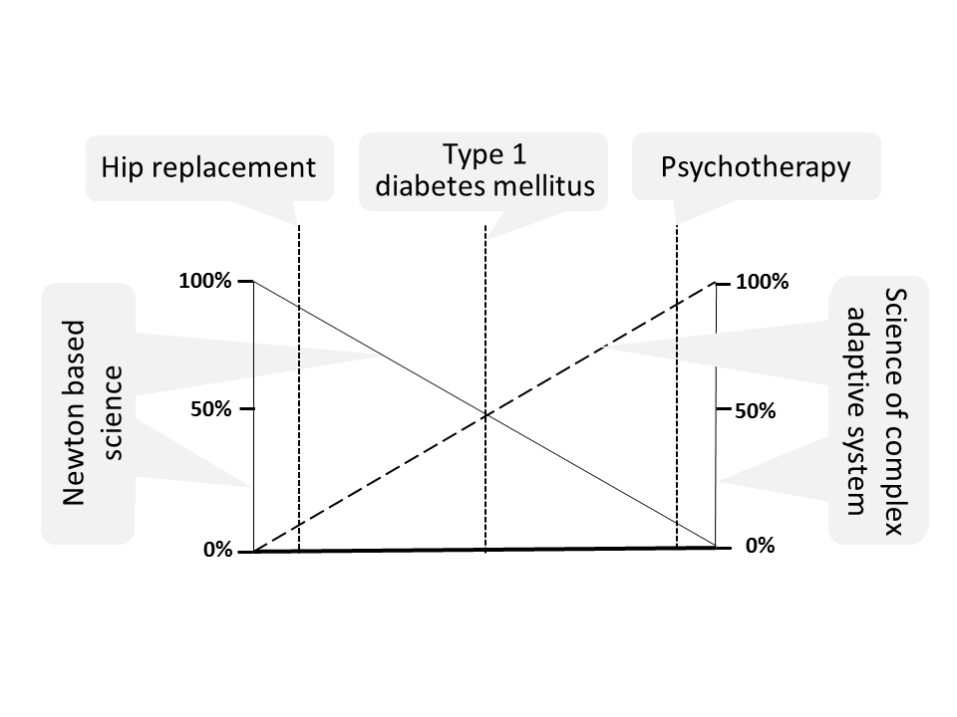
Traditional and person-centered care Comparison of traditional medicine and person-centered care. The ordinate on the left-hand side expresses Newtonian science as it is customary in traditional medicine. The right-hand side represents thinking used in complex adaptive systems (CAS). Each diagnosis and treatment must always consider both factors: For hip replacement, methods of conventional medicine are predominant, and for psychotherapy, methods are based on CAS. Yet, in each case, the other factor also applies to some degree. Interestingly, the example of Type 1 diabetes mellitus is approximately in the middle. The patient must understand the physiology of glucose and insulin metabolism and must be able to measure blood glucose and inject insulin. Ultimately, however, he or she must feel that meticulous self-treatment serves her or his purpose best because it leads to the best possible future. This insight results from processes related to a CAS.

Many acute and chronic conditions of the physical body are appropriately treated by surgery or by drugs, as supported by empiric evidence and by the current practice of medicine. Such interventions in the BGP may be essential when a treatment is a lifesaving or when it may prolong a meaningful state of health. Today, the efficacy of this approach is demonstrated by the well-recognized successes of clinical research and healthcare, including evidence-based medicine. Yet, in every case, surgery or drug treatment is applied not simply to a biological organism, but specifically to an entire human being that responds with both of his or her potentials. This is illustrated in Figure 4. Insertion of a hip prosthesis concerns primarily the BGP and is therefore categorized as Newtonian science. On the other hand, psychotherapy deals mainly with the PAP and therefore reflects complexity science. However, both are not mutually exclusive. Recovery from a hip operation and rehabilitation strongly involve the PAP. Analogously, the physical symptoms of patients undergoing psychotherapy require adequate attention to the individual’s BGP. Interestingly, self-care of patients with Type 1 diabetes mellitus can be regarded as based on both the BGP and PAP, almost in equal parts. Measurement of blood glucose, adherence to an adequate diet, physical activity, and the injection of insulin concern the BGP and reflect Newtonian science. Knowledge of the glucose and insulin physiology and disciplined adherence to treatment, on the other hand, require a correspondingly developed PAP that reflects complexity science. In addition, appropriate care must make sense. This requires that patients and healthcare providers must equally respect the causality of Newtonian science and the rules of systems theory necessary to deal with a CAS.

Diseased persons, especially those with chronic conditions, when neither surgery nor drug therapy is indicated, should receive particular attention to their health as a CAS. In this situation, the physician must consider all five components and all ten interactions of the system [6]. As a result, some critical aspects may be discovered that can be discussed with the patient. Yet, as is typical of considering a patient’s health as a CAS, the adaptive response to and the further evolution of the CAS will have to come from the patient himself or herself and all manipulations will disturb this process and should be avoided. Depending on the further evolution of the system, the condition of a patient may improve, remain stable, or even deteriorate. A favorable outcome for the patient as a “system” may be influenced by many different factors. In every case, a patient-doctor relationship based on mutual confidence, together with empathic attention to the personhood of the patient, is helpful but may not suffice. Some patients may require formal psychotherapy [11] or be helped by selected procedures or even complementary medicine [13]. Obviously, the favorable evolution of a CAS cannot be predicted and needs frequent follow-up and adjustments in interventions. Efforts for further scientific investigation of CASs in healthcare should now receive high priority (Table 1).

Growth of the Personally Acquired Potential

**Figure 5:**
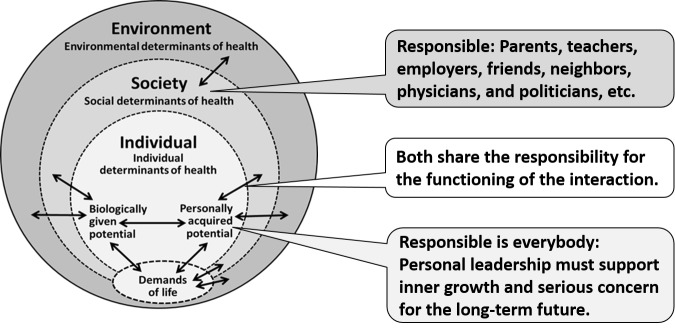
Responsibility for health Responsibility for health is shared by members of society and by the personal acquired potential of each individual and by members of society. Both components of the model have to fully contribute their own part. Well-functioning interactions among the two components are critical for an effective sharing of responsibility.

The concept that development of one’s own personality is an individual’s personal responsibility is well recognized. The social setting, including the responsibility that resides with the political system, is also of importance [14]. It may render personal growth easier or more difficult (Figure 5). In addition, some techniques for evolution as an individual, such as meditation or prayer, have been available for a long time [15-17]. In recent years, techniques of mind-body medicine have been developed and appear to enhance personal growth, result in more than average happiness, and are associated with longevity [18-21]. In this context, it is also of interest that mindfulness broadens awareness and builds eudaimonic meaning [15]. Translated into the framework of the Meikirch model, these observations support the idea that more attention to the PAP will have important beneficial consequences for the health of individuals

Interprofessional and Intersectoral Cooperation

**Figure 6:**
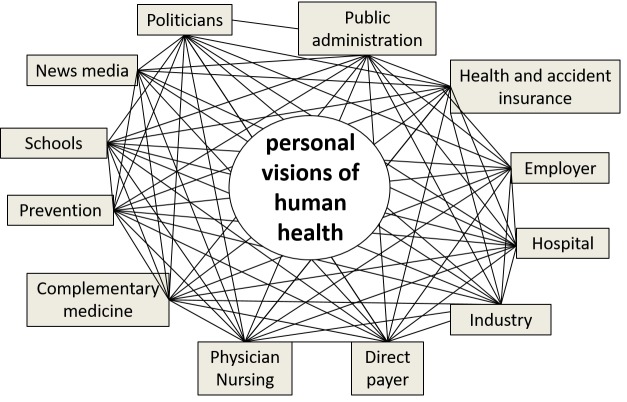
Interprofessional and intersectoral cooperation is improved by shared objectives Interprofessional and intersectoral cooperation functions best when all the involved persons serve the same objective. In the case of healthcare, this is difficult because personal visions of health vary from individual to individual. Thus far, health has been an ill-defined concept, and as a result, the concept has not been unifying. Once the Meikirch model is placed in the center, all participants in the health care system are able to work toward the same objectives, i.e., the health of patients.

Today’s healthcare has become complex and requires cooperation among many professionals in different fields [22-23]. For many reasons, this complexity often leads to misunderstandings that may be very costly. Physicians and nurses, for example, sometimes do not agree on their respective competencies. Moreover, different specialties may set different priorities, and administrators may be more concerned with costs than with a patient’s health. Figure 6 illustrates the various professionals and institutions that are concerned with health and shows the multitude of possible interactions. All involved persons have developed their own individual vision of human health; consequently, they cannot agree easily on common goals. This situation may explain some of the many conflicts among persons who share responsibility for some aspects of healthcare. It is our hypothesis that the Meikirch model offers much more precise objectives for the division of labor in the interest of a joint purpose. As a result, the details of conflicts of interest may become more transparent and negotiable. The Meikirch model will thus allow a more rational exchange of opinions about every problem, possibly leading to better solutions and resulting in significantly improved cooperation.

The PAP is small after birth (Figure 2) and increases rapidly thereafter. During adolescence, young people start to assume responsibility for their lives. Many of them, however, have never been taught that they are also responsible for continuously investing in their future health. Discouraging smoking, physical inactivity, obesity, drug abuse, and sexually transmitted diseases is perceived as an inconvenience. Yet, in our personal experience, the Meikirch model is easily understood by people of diverse backgrounds. For instance, teaching about the model has been successful in villages of indigenous people in India. Assessment of their health-supporting behavior after the introduction of the Meikirch model revealed an impressive improvement [24]. It is our hypothesis that the need for costly health care may be significantly reduced by appropriate and repeated teaching about the Meikirch model and its ramifications, including all important aspects of health. This should start in kindergarten and elementary school and continue throughout life. Realization of this postulate is a responsibility of society (Figure 5). Due to better health literacy and competency, healthcare costs are expected to diminish appreciably. Obviously, health information would have to have an unbiased and a trustworthy origin and, therefore, cannot come from industry. Once patients in our health system are accustomed to the Meikirch model, we presume that the patient-physician interaction might also benefit, and adherence to the advice of the physician is likely to improve.

Public Health

Society must assume responsibility for the health of its population in many ways [25]. Supervision of epidemics, care for waste and sewage disposal, and administration of proper food handling are just a few examples of essential, larger undertakings. In addition, the organization of healthcare, including ambulatory and hospital medicine and health insurance, is also a task of society. One of the most important points is a functioning relationship between society and individuals (Figure 5). In this sphere, hidden conflicts of interest must be made visible in order to induce procedures to solve them. This is relevant in order to maintain the trust of the public and patients. Currently, large companies try to stimulate demand for their products by direct to consumer advertising as well as influencing governments and physicians. Unfortunately, these trends have undermined the trustworthiness of advice by physicians and public health experts. The results may be seen, for example, in poor patient compliance and unsatisfactory vaccination rates. Trust is the most precious commodity in healthcare and therefore needs close attention.

Payment Modalities Must Place Health at the Center!

Until recently, health needs have been the single most important concern physicians and nurses shared with their patients. This led many healthcare workers to consider their profession as a calling. Yet, due to rising healthcare costs, economic considerations, including various payment systems, have become equally (or even more) important as health itself [26-27]. This has become particularly apparent since the introduction of value-based payments. Thus, the question has now arisen as to whether healthcare with a concern for patients (patient-centered healthcare) is a calling or a business. When analyzed according to the Meikirch model, this question has two answers (Figure 4): Therapeutic procedures to correct or compensate for defects of the BGP successfully use Newtonian science predominantly (Table 1) and may, in fact, be handled as a business. Outcome per costs can be measured and calculated. In contrast, care for the development of a PAP requires the application of complexity science based on the rules of a CAS (see above: Complex Adaptive System and Table 1). Results can be as successful as those for the treatment of the BGP, but different methods are used, such as sustained and caring attention to the patient and/or psychology focused on the patient’s PAP. This might require new metrics for health services and health outcomes to reflect the attention to the PAP.

A further example is a movement to promote integrative medicine, as proposed by the Academic Consortium for Integrative Medicine and Health. This movement reaffirms the importance of the relationship between practitioner and patient, focuses on the whole person, is informed by evidence, and makes use of all the appropriate therapeutic and lifestyle approaches, healthcare professionals, and disciplines to achieve optimal health and healing [13]. The National Center for Complementary and Integrative Health has recently proposed a strategic plan to explore the science of complementary and integrative health [28]. Both efforts are fully compatible with the concept of the Meikirch model: Integrative medicine requires high expertise but less technology; outcomes depend on the sustained participation of the patient and his or her PAP and are less predictable, in agreement with the concept of a CAS. Such settings are incompatible with value-based payment and other performance-based payment systems.

The Meikirch model, based on human nature with its two potentials, BGP and PAP, calls for new philosophical and economic considerations. Value-based healthcare, as introduced by Michael Porter and associates, neglects the patient´s PAP and the science of CASs. Interestingly, this inadequacy has been recognized and has led to movements that try to place the human nature of patients at the center of healthcare again. Examples are the “European Society for Patient Centered Healthcare” [29] and the Academic Consortium for Integrative Medicine and Health,” as mentioned above [13]. It is hoped that the Meikirch model will be helpful in the development of new and more appropriate payment systems.

## Conclusions

In this paper, a selection of six important consequences of the Meikirch model, when applied to our present healthcare system, are explored. The conceptual framework of the model is still a hypothesis and now requires testing and confirmation by experimental research. Thus far, most explored scenarios have revealed new perspectives and consequences of the model that deserve to be evaluated. 

If health truly is the main purpose of healthcare, it is surprising that in the past physicians have not made more efforts to illuminate an understanding of health. The Meikirch model has been built on evidence available at the time and on our personal experiences and insights as healthcare professionals. A model of individual health was first proposed in 2005 [30]. Thereafter, few contributions were made until a model of combining individual health with public health was proposed in 2014. When the Meikirch model was subsequently projected on various aspects of healthcare, interesting and pertinent consequences were revealed. We assume that even more consequences will become apparent with time. These experiences document that health may no longer be regarded as a “blind spot”.

An important consequence of the model may relate to healthcare financing. Table 1 and Figure 4 illustrate that the two different approaches to health--Newtonian science and complexity science--are always combined in clinical practice, the two approaches are fundamentally different, and, in every patient, both have to be taken into account simultaneously to varying degrees. The dual nature of health, with contributions by the BGP and the PAP, suggests that the financing of healthcare should be adapted correspondingly in order to avoid inappropriate incentives. It is evident that value-based financing is suitable only for procedures that are based mostly on Newtonian science. In contrast, patients with a strong component of health as a CAS render value-based healthcare irrelevant. In addition, new methods for handling intermediary cases need to be created. In view of the unbalanced increase in healthcare costs (favoring procedures), a more appropriate type of remuneration for health care based on both potentials (BGP and PAP) should be developed.

We believe that attention should now be paid to focusing healthcare on each individual patient as a person. The Meikirch model presents health literacy and responsibility for health in a new light. The PAP and the character of health as a CAS are new touchstones that should stimulate more personal responsibility for health. In particular, attention to personal growth may now be a goal for individuals, medicine in general, and public health. The postulates of the Meikirch model are fully in line with the theory and practice of evidence-based medicine, person-centered healthcare, and integrative medicine. The Meikirch model may, for example, give complementary medicine a new rationale: its procedures are mostly nondirective and often associated with personal attention to the patient and his or her motivations. Such features may induce the CAS of the patient to evolve autonomously toward a state of better health. It is our hypothesis that once the healthcare system has included all changes proposed by the Meikirch model, not only may the health of the population improve substantially but, at the same time, healthcare costs may also decrease appreciably.

The Meikirch model is at the core of healthcare. It is a new conceptual framework of health that may touch on many, if not on all, aspects of medicine and public health. The model may, therefore, be expected to induce gradual changes in healthcare that correspond to a paradigm shift. The authors invite the healthcare community to acquaint themselves with this model in greater depth and explore its potential and all of its ramifications.

## References

[1] Fineberg HV (2012). A successful and sustainable health system--how to get there from here. N Engl J Med.

[2] Rosenthal DI, Verghese A (2016). Meaning and the nature of physicians’ work. N Engl J Med.

[3] Porter ME, Lee TH (2015). Why strategy matters now. N Engl J Med.

[4] Committee on Economic, Social and Cultural Rights (2016). Report on the Twenty-Second, Twenty-Third and Twenty-Fourth Sessions. IV. General Comment No. 14 (2000): The right to the highest attainable standard of health (art. 12 of the International Covenant on Economic, Social and Cultural Rights). United Nations.

[5] Bircher J, Kuruvilla S (2014). Defining health by addressing individual, social, and environmental determinants: new opportunities for health care and public health. J Public Health Policy.

[6] Bircher J, Hahn EG (2016). Understanding the nature of health: New perspectives for medicine and public health. Improved wellbeing at lower costs. F1000Research.

[7] Bircher J, Hahn EG (2016). Applying a complex adaptive system’s understanding of health to primary care. F1000Research.

[8] Peters DH (2014). The application of systems thinking in health: why use systems thinking. Health Res Policy Syst.

[9] Lansing JS (2003). Complex adaptive systems. Ann Rev Anthrop.

[10] Lamb SE, Hansen Z, Lall R, Castelnuovo E, Withers EJ, Nichols V, Potter R, Underwood MR; Back Skills Training Trial investigators (2010). Group cognitive behavioural treatment for low-back pain in primary care: a randomised controlled trial and cost-effectiveness analysis. Lancet.

[11] Balint M (1954). Training general practitioners in psychotherapy. Br Med J.

[12] Antonovsky A (1987). Unraveling the mystery of health - how people manage stress and stay well. http://www.amazon.com/Unraveling-Mystery-Health-Behavioral-Science/dp/1555420281/ref=sr_1_1?s=books&ie=UTF8&qid=1488421341&sr=1-1&keywords=Unraveling+the+mystery+of+health+-+how+people+manage+stress+and+stay+well.

[13] Ring M, Brodsky Brodsky, M M, Low Dog T, Sierpina V, Bailey M, Locke A, Kogan M, Rindfleisch JA, Saper R (2016). Developing and implementing core competencies for integrative medicine fellowships. Acad Med.

[14] Wilkinson R, Pickett K (2009). The Spirit Level: Why Equality Is Better For Everyone. https://www.amazon.com/Spirit-Level-Equality-Societies-Stronger/dp/1608190366/ref=tmm_hrd_swatch_0?_encoding=UTF8&qid=1488437774&sr=1-1.

[15] Garland EL, Farb NA, R. Goldin P, Fredrickson BL (2015). Mindfulness broadens awareness and builds eudaimonic meaning: a process model of mindful positive emotion regulation. Psychol Inq.

[16] Cherkin DC, Sherman KJ, Balderson BH, Cook AJ, Anderson ML, Hawkes RJ, Hansen KE, Turner JA (2016). Effect of mindfulness-based stress reduction vs cognitive behavioral therapy or usual care on back pain and functional limitations in adults with chronic low back pain: a randomized clinical trial. JAMA.

[17] Hölzel BK, Lazar SW, Gard T, Schuman-Olivier Z, Vago DR, Ott U (2011). How does mindfulness meditation work? Proposing mechanisms of action from a conceptual and neural perspective. Perspect Psychol Sci.

[18] Chopik WJ, Kim ES, Smith J (2015). Changes in optimism are associated with changes in health over time among older adults. Soc Psychol Personal Sci.

[19] Danner DD, Snowdon DA, Friesen WV (2001). Positive emotions in early life and longevity: findings from the nun study. J Pers Soc Psychol.

[20] Fredrickson BL (2009). Positivity. http://books.google.com/books?hl=en&lr=&id=sECwrAYb9wUC&oi=fnd&pg=PA3&dq=Positivity&ots=3kykNqgaPI&sig=faAcFbb3mzxVXqa6XL7wWhonpD4#v=onepage&q=Positivity&f=false.

[21] Gulliksson M, Burell G, Vessby B, Lundin L, Toss H, Svärdsudd K (2011). Randomized controlled trial of cognitive behavioral therapy vs standard treatment to prevent recurrent cardiovascular events in patients with coronary heart disease: Secondary Prevention in Uppsala Primary Health Care project (SUPRIM). Arch Intern Med.

[22] de Savigny D, Adam T (eds) (2017). Systems Thinking for Health Systems Strengthening. World Health Organization.

[23] Bircher J, Wehkamp KH (2011). Health care needs need to be focused on health. Health.

[24] Samal S, Mohanti D, Born E, Bircher J (2017). Teaching of health with the Meikirch model to indigenous people improves their health-supporting behavior: A pilot study. Med J DY Patil Univ.

[25] Marmot M, Wilkinson RG (2006). Social Determinants of Health, Second Edition. Social.

[26] Porter ME, Larsson S, Lee TH (2016). Standardizing patient outcomes measurement. N Engl J Med.

[27] Porter ME, Teisberg EO (2006). Redefining Health Care. Creating Value-Based Competition on Results. Redefining.

[28] NIH. National Center for Complementary and Integrative Health (2016). NCCIH 2016 Strategic Plan. http://nccih.nih.gov/about/strategic-plans/2016.

[29] Miles A, Asbridge JE (2013). The European Society for Person Centered Healthcare. Eur J Pers Centered Healthc.

[30] Bircher J (2005). Towards a dynamic definition of health and disease. Med Health Care Philos.

